# Striking a balance: Stakeholder perceptions of risk in horse racing

**DOI:** 10.1111/evj.14561

**Published:** 2025-07-07

**Authors:** Jessie McCarthy, Heather A. Cameron‐Whytock, Euan D. Bennet

**Affiliations:** ^1^ School of Biodiversity, One Health, and Veterinary Medicine University of Glasgow Glasgow UK; ^2^ School of Veterinary Medicine University of Central Lancashire Lancashire UK

**Keywords:** horse, horse racing, horse welfare, industry perception, risk factors, Thoroughbred racing

## Abstract

**Background:**

Thoroughbred racing is a major industry, and in recent years public concerns about equine safety have become more prominent, particularly in relation to on‐track injuries and fatalities. This has challenged the industry's social licence to operate (SLO).

**Objectives:**

To investigate and elucidate how United Kingdom and Irish racing stakeholders perceive risks to racehorses on race day and how those risks should be managed.

**Study Design:**

Qualitative analysis of stakeholder perspectives using a constructionist approach.

**Methods:**

Twelve stakeholders from veterinary, communication, and regulatory sectors within racing in the United Kingdom and Ireland were interviewed. Semi‐structured interviews were analysed using reflexive thematic analysis.

**Results:**

Three key themes were developed ‘Managing Risk, or Managing the Message?’, ‘The Balance between Tradition and Progress on Reducing Risks’, and ‘Attributing Responsibility and the Public Disconnect’. Participants framed risk according to public misunderstanding and effective communication, while others placed increased emphasis on welfare risks and proactive mitigation measures. Some participants viewed ‘accidents’ as unavoidable, others believed that more could be done to minimise avoidable risk. Attribution of responsibility was directed towards various industry stakeholders including trainers, jockeys, regulators and the public. Interviewees noted the industry to be rooted in tradition and slow to adapt to changing expectations.

**Main Limitations:**

Familiarity between the interviewing researcher and some participants may have encouraged open discussion but could have influenced how responses were framed.

**Conclusions:**

Stakeholders framed risk in ways shaped by public expectations, culture, tradition, and lived experience. There is shared concern for equine welfare and a desire for improved safety measures in conjunction with the management of public perceptions. For a sustainable future, internal divisions must be resolved, shared goals established, and proactive engagement with science pursued to safeguard equine welfare and sustain public support.

## 摘要

**背景:**

纯血马赛马是一项重要产业，近年来，公众对马匹安全的关注日益增加，尤其是在赛道受伤和意外死亡事件方面。这种关注已对该行业的社会运行许可(social license to operate， SLO)构成挑战。

**目标:**

研究并阐明英国和爱尔兰赛马利益相关者如何看待比赛日马匹所面临的风险，以及应如何管理这些风险。

**研究设计:**

采用建构主义方法对利益相关者的观点进行定性分析。

**方法:**

对来自英国和爱尔兰赛马界的兽医、传播和监管领域的 12 位利益相关者进行了访谈。对半结构式访谈内容采用反思性主题分析法进行分析。

**结果:**

研究归纳出三个重要主题:“是管理风险，还是管理信息?”，“在降低风险中寻求传统与革新的平衡”， 以及“责任归属与公众脱节”。部分受访者从公众误解和有效沟通的角度来分析风险，也有部分受访者更强调福利风险以及主动的防范措施。一些受访者认为“事故(accidents)”不可避免，另一些受访者则认为仍有更多措施可被采取去尽量减少可避免的风险。在责任归属方面，受访者认为责任应涉及各个从业利益相关者，包括教练、骑师、监管者及公众。受访者指出赛马行业根植于传统，因此在适应不断变化的社会期望时相对缓慢。

**主要局限性:**

研究者与部分受访者之间的熟悉关系可能有助于坦诚对话，但也可能影响了回答的表述方式。

**结论:**

利益相关者对于风险的定义受公众期望、文化、传统和自身经历的影响。受访者普遍关心马匹福利，并希望在管理公众认知的同时加强安全措施。为了行业可持续发展，从业者须解决内部分歧，确立共同目标，并积极引入科学依据，来保障马匹福利与维系公众支持。

## 1 INTRODUCTION

Thoroughbred racing, the United Kingdom's second most attended sport contributes £3.7 billion grossly to the national economy.^1^, ^2^ However, public concerns for equine safety have intensified in recent years, as injuries and fatalities on track are seen to be incompatible with commitments to animal welfare.^3^, ^4^ Central to this devolution is the concept of a ‘social license to operate’ (SLO), an implicit agreement between society and an industry or organisation that allows its activities based on shared values, trust, and legitimacy.^5^ Historically, racing maintained its SLO by emphasising the care and affection afforded to racehorses, the sport epitomising the human‐animal partnership.^6^

British Horseracing Authority (BHA) figures show horse mortality on UK racetracks has decreased by a third in the last 20 years. Equine injuries and fatalities occurred in 0.18% of races in 2023 with a 5‐year average of 0.20%.^7^ Despite these advancements, a culture of risk acceptance exists amongst the racing community; fatalities are often thought of as ‘accidents’—fluke events due to ‘unavoidable risks’ in a well‐regulated industry, which suggests that a disconnect exists between incidents and the conditions that induced them.^3^, ^5^, ^8^

Risk perception plays a role in the divide between public stance and industry opinion. Irrespective of safety improvements, the public remains concerned about equine safety. Patterson and Hodge reported that double the proportion of participants (84.5%) expressed ethical concerns about the risk of injury to horses in racing compared with 40.3% who had similar concerns about the risk of injury to human athletes in other competitive sports.^9^ Effective risk management in racing involves not only identifying and mitigating risks but also communicating these efforts.^10^ Stakeholders play a vital role in all phases of the risk management process; understanding and integrating stakeholder perceptions of risk has demonstrated increased success of remediation efforts within other sectors.^11^ Integrating risk perception analysis is crucial to identifying drivers and barriers to sustainable industry change.

This study addresses a gap in understanding of how key stakeholders perceive and manage race day risk to horses. While public concerns are well‐documented, the perspectives of veterinarians, media professionals, and governance officials remain unreported in the literature. These stakeholders are uniquely positioned as authorities on public expectation, regulatory action, and equine care. By conducting semi‐structured interviews and situating these findings within the broader context of equine welfare and risk management, this study explores the research question: *How do stakeholders perceive and approach risk on race day?* By examining how these individuals frame risk and responsibility, this study aims to examine the institutional and cultural context in which decisions around risk are made.

## 2 MATERIALS AND METHODS

### 2.1 Qualitative approach and research paradigm

Qualitative methods were chosen to enable a deeper understanding of perceptions that would not be possible with a quantitative paradigm. This research was conducted using the lens of social constructionism, which recognises that people, including researchers, construct meaning through their own situated perspectives. As such, findings are shaped by the researcher's experiences and represent one possible interpretation of reality, rather than a single objective truth.

Semi‐structured interviews were selected to capture rich contextual data based on participants lived experiences, with flexibility to explore answers and ask follow‐up questions.^12^, ^13^, ^14^, ^15^, ^16^, ^17^ This approach allows participants' views to directly shape the development of themes, meaning the analysis is in their accounts rather than producing generalisable findings. One author (JM) conducted all interviews with participants during June 2024.

### 2.2 Positionality statement by JM

Growing up on a Thoroughbred stud farm and as a regular race attendant has allowed me an ‘insider's’ perspective on the racing industry, while involvement in other equine sport industries has broadened my views. My industry connections have fostered participant trust, essential for openness, though this immersion may introduce biases.^18^ To reflect on this, I kept written notes after each interview and discussed my interpretations with the other researchers in order to better understand how I relate to this research.^19^

In some instances, my training in evidence‐based practice and familiarity with industry perspectives led to moments of dissonance. These moments prompted me to consider whether I was viewing responses through an academic or experiential lens. In some cases, this prompted me to reword or rethink early theme labels to better reflect participants' own language and perspectives.

### 2.3 Participant selection and recruitment

Non‐probabilistic purposive sampling was used to intentionally select participants with relevant expertise.^20^ Twelve interviews were conducted in total. This number was informed by the concept of information power, in which the relevance of participants and richness of data influence how many interviews are required.^21^ The researchers' familiarity with the topic and concurrent analysis also supported this decision. Participants were recruited in May and June of 2024. On first contact, individuals were provided with a summary of the project and invited to express interest in participating. On agreement, participants were provided with a privacy notice and a consent form. They were informed of their right to withdraw from the study at any time. Nine participants were contacted through previously known contacts or via publicly available contact details, with an additional four participants recruited through snowball sampling. Inclusion criteria focused on individuals working in regulatory, communication, and health or welfare‐related roles within the United Kingdom and Irish racing industries, including veterinarians, media professionals, and industry board members. These groups were selected for their relevance to how risk and welfare are interpreted, communicated, and managed across different levels of the industry. It must be noted that veterinarians were included due to their direct involvement in equine health and oversight on race days. However, health is only one component of animal welfare, and vets might not cover all aspects of welfare expertise. Additionally, while race day risk is heightened in jumps racing and several interview questions focused on this discipline, the study did not specifically differentiate between National Hunt and Flat racing. Participants often referred to both forms interchangeably unless otherwise stated.

### 2.4 Data collection

One author (JM) conducted all interviews with participants during June 2024. Reflexivity was maintained through ongoing reflection on the researcher's role in shaping the data.^22^, ^23^, ^24^, ^25^

Three pilot interviews were carried out to refine the question guide, which is available in Data S1. Interview topics included participants backgrounds, opinions on risk, and views on safety measures. Researcher goals were not discussed during the interviews to avoid response bias. Each interview was digitally recorded via Zoom.^26^ All participants gave written and verbal consent to digitally record the interviews and were made aware of their right to halt the recording at any point throughout the interview. They were also informed of the secure storage of the recording files and the anonymisation of transcripts to protect their identities. No other persons except the interviewer and interviewee were present throughout each interview.

### 2.5 Data analysis

The audio recordings were transcribed verbatim using Zoom transcription software and manually checked against recordings for accuracy and to remove identifying features. Visual and audio recordings were subsequently deleted, retaining only anonymised transcripts for data analysis. Transcripts were imported into Nvivo^1^ for reflexive thematic analysis, following Braun and Clarke's six stage method (1. Familiarisation with the data, 2. Coding, 3. Generating initial themes, 4. Developing and reviewing themes, 5. Redefining, defining, and naming themes, 6, Writing up). Reflexive thematic analysis is a form of thematic analysis that places an emphasis on researcher reflexivity, views the practice of thematic analysis as inherently subjective, and assumes meaning is shaped from the researcher's interaction with the data.^12^, ^13^, ^14^ One researcher conducted the analysis independently, with themes refined over multiple rounds of coding, reflection, and consultation with the other researchers. The researcher actively engaged with the data through repeated reading and coding, using an inductive, semantic approach. This means themes were developed from the data itself, with focus on the explicit content of participants' responses rather than latent assumptions.

## 3 RESULTS

Twelve interviews were completed, each taking between 26 and 51 min. Six women and six men were included in this study. Participants were split evenly across three professional categories, four veterinarians, four industry board members and four race media professionals. Five participants were primarily involved in Irish racing, and seven in UK racing. Eleven of twelve participants came from an equestrian or rural background, with industry experience ranging between six and thirty‐plus years. Participants are labelled, media (M), vet (V) and regulator (R) and assigned a number between 1 and 4. Via Thematic analysis three key themes were created: ‘Managing Risk, or Managing the Message?’, ‘The Balance between tradition and progress on reducing risks’ and a third theme of ‘Attributing responsibility and the public disconnect’. Each theme is explored below.

### 3.1 Managing risk, or managing the message?

This theme explores how stakeholders opine and position risk in relation to public perception and the practical realities of race day. Discourse painted risk as something to be explained, minimised, or normalised.

Some stakeholders focused on how the public interprets risk, framing a public misperception. These stakeholders described the sport as safer than ever. Low fatality rates were cited as a point of pride. One stakeholder noted,

We can't hide, or we shouldn't hide fatality rates. We should be proud that they are as low as they are. (R4)

A participant involved in media, emphasised the importance of public relations and improving public understanding,

I think it's ultimately a PR game, a communications game. I don't think we can hide from those realities, sometimes harsh realities, but we have to deliver those realities in a way that's sympathetic to the outlook of the people we are delivering it to … we have to explain, we have to help people understand and appreciate why certain things happen, the reasons why we deal with tough situations in a certain way, and look, just essentially hope that people will get it, and some people never will. (M4)

Another participant expressed that public perception is often shaped by a lack of factual understanding:

The chasm between perception and reality when it comes to racing is huge, and it's understandable that people, if they don't have the facts, and there's a vacuum, they're going to fill the vacuum with perceptions. It has to be about education; it has to be about facts. (M2)

Several participants felt that public concerns for equine safety on race day were misplaced, the real issues were at home, backstage. They emphasised the minimal time spent racing relative to the overall time horses spent in training.

They're racing for 5 half days, you know, and they're actually only racing for 5 minutes on that day, and compared to the overall risk, or what happens in the rest of their life, or for that year, or those 3 or 4 years that they are in training, you know it's probably relatively small. (V1)

The reality is that the horses are in better care on a racecourse for me than anywhere else. (V3)

Stakeholders acknowledged that risk is an inherent part of the sport. Accidents were unanimously perceived to be inevitable. One participant stated,

We consciously do it with that element of risk which is ultimately unavoidable to a hundred percent. (M1)

Veterinarians were particularly aware of the risk on race day, these participants expressed increased consideration for horses at the races both on and off the track:

Every participant that enters through the stable gate that day is potentially at risk. (V2)

A balancing act was observed between accepting the inevitability of inherent risk associated with race day and striving for improved safety. One participant expressed this sentiment:

We always have to operate within the confines and within the knowledge of that fact that it carries a level of risk which will never be eradicated entirely. (M2)

Stakeholders negotiated a spectrum of views regarding risk, acknowledging tangible mitigation measures are required while some placing emphasis on misperception as a cause of concern over risk. Stakeholder opinions were nuanced reflecting the complexity of the issue.

### 3.2 Balance between tradition and progress on reducing risks

Proactive solutions to risk were a key focus amongst all interviewees. However, entrenched practices and reluctance to change were consistently cited to hinder implementation of effective risk management strategies. Progress and advancement were consistently anchored to the past. Racing's proud history was mentioned frequently, one participant exclaiming that racing was very much part of ‘Merry old England’ (V1).

Providing the public with accurate information and injury statistics was highlighted as important facets of risk engagement. One participant noted the importance of having the correct information in forming opinions,

It's about nailing your evidence and stats and saying, this is the reality. This is where fatality rates really are. This is what we've done to reduce them. This is how we've reduced them. This is how we continue to do that further. You've got to. When in that situation, you just got to rely on simple, basic facts. That sounds so glib. But it's really about, how do you overcome that fact that people have a natural distrust. (R1)

This emphasis on transparency was often linked to an appetite for tangible improvements. Some interviewees felt identifying and reducing avoidable risks was a first step.

A really key word in the approach to risk that the sport takes is looking at what risk can be reasonably avoided and what can be done to mitigate that. (M2)

Yet, the same participant later noted the challenge of enacting change within the industry.

We are a sport that leads itself to ingrained attitudes in some way. Quite a lot of our sport is handed down from one generation to the next and we don't always welcome outsiders potentially, or people challenging our views. (M2)

This resistance to change was linked to a broader mentality within the industry. One participant noting,

Racing has always had this, ‘we're above everything, leave us alone’ mentality. (V2)

V3 historically described a similar industry resistance:

We've come from a place where we were a bit reticent about even talking about this, you know, there was a time when we wouldn't talk about openly, about stats or things we were doing, the ethos was that we'll just add momentum to the story. The story's there though and people are gonna be talking about it anyway. So, we need to be on the front foot telling the positive story as an industry that I think we've got to tell. (V3)

Participants articulated a clear desire to improve safety, but efforts to do so were often shaped by an industry culture routed in tradition.

### 3.3 Attributing responsibility and the public disconnect

This theme reveals how stakeholders assign and distribute responsibility for race day risks. Frequently, responsibility was directed towards other members of the industry or attributed to cultural factors. Stakeholders identified a challenge in not only addressing the internal and external factors contributing to race day incidents but also in navigating an evolving landscape of public opinion and expectations. Participants often identified other stakeholders within the industry as responsible when discussing risk. Jockeys, trainers, owners, and horses themselves were often cited as factors in race day incidents. When asked directly about whether horses were injury‐prone, participants pointed to equine biology and behaviour. Thoroughbreds were thought to inevitably injure themselves.

You often wonder how the leg doesn't snap even more often. But I mean they run in the wild, they run in the field, they run at stud. It's what they do. The mechanics of the horse are not good, it's not like a formula one car. (M3)

So this is where we talk about paddock accidents as the get out of jail card for racing, because the horse himself, unbridled, unlimited will gallop to the edge, will sustain injuries in doing so, particularly because in those kinds of environments, the surface they are galloping on isn't as manicured, and prepared as the ones we ask them to gallop on at the race course. (V4)

Blame was also said to be placed on racecourse conditions rather than addressing underlying issues with the horses themselves. One participant described how trainers might shift blame to external factors:

It gets injured, then the tracks are to blame, they blame the ground, they'll blame the racecourse, whereas, you know the horse came with a problem. (R3)

Regulatory bodies were also scrutinised for their perceived lack of proactive measures. One participant was particularly candid in their critique of the British Horse Racing Authority (BHA),

I don't think the BHA deserve any pat on the back …. They waited until we had a ******* complete disaster, and the house nearly burnt down before they put in a fire extinguisher. (M1)

Another participant expressed frustration at the industries reactive rather than proactive approach to welfare, which they felt led to poorly thought‐out decisions,

Here we have leaders of the industry, saying, because the welfare train is pulling out of the station, lets jump on this welfare train without giving it the slightest bit of thought, and I cannot believe that's the kind of thing you are dealing with you know. (V4)

Participants spoke of internal industry divisions. One media professional felt that conflicting voices and internal divisions often undermined the sports' own communications.

I believe you need to get the sport lined up and reading from the same hymn sheets. It's very difficult to reach a wider audience with one campaign and then you have your messaging undermined by people from within the sport. (M2)

Alongside internal attributions of responsibility, some participants felt public misunderstanding of racing was not entirely a product of racing miscommunication but instead reflected a wider cultural chasm. Speaking as industry insiders, participants positioned their understanding of risk as pragmatic and having evolved from a lifetime of hands‐on experience.

Look at the background I come from, a farming background, an agricultural background, it's a bit more pragmatic when it comes to animals. Dare I say, a lot of racing's audience doesn't have that background. They have a love and an appreciation for the animals, but maybe might be a little bit removed of the on the ground realities of dealing with them, of training them, of the difficulties involved in what is done with them. So, I'd like to think that I have a pragmatic view of what we're doing you know, and I suppose I'm a bit more conditioned to some of the tougher realities of what we do. (M4)

Generational differences and public scepticism was raised. One participant lamented the impact of public cynicism on the industry:

Some people are just so cynical; they just don't believe anything you're telling them and racing probably suffers to some degree from that natural scepticism of people. (R1)

Participants noted the public behaves as an everyday activist and has become increasingly emboldened to question existing power structures. A mentality that was often thought to be fuelled by social media and changing engagement with news. A trend of increased engagement and scrutiny was touched upon:

‘it's since the institutions have fallen so the Church used to be extremely powerful, and it got ridiculed out of existence … authority figures generally are derided, ridiculed, disrespected, you know, and that's sort of empowering everybody to be, you know, to be in charge ….’ ‘That sort of flash mob mentality about news stories. I haven't really figured out where it fits in all of this. But I think it's a social media thing of “I have an opinion on how you live your life, and I am free now to tell you how to live your life”’. (V4)

## 4 DISCUSSION

This study finds nuance in risk perception amongst influential stakeholders in the United Kingdom and Irish horse racing industry. Three key themes: ‘Managing Risk, or Managing the Message?’, ‘The Balance between tradition and progress on reducing risks’, ‘Attributing responsibility and the public disconnect’ were formed.

Stakeholders acknowledged the inherent risks in racing but varied in how they framed this risk, describing the industry as increasingly welfare focused. When asked, some emphasised risk in terms of management, others focused on public perception, and others on attribution. While risk mitigation was a common concern, participants often felt public perception must be managed in conjunction.

When discussing perception in relation to risk, participants emphasised low fatality rates and risk management practices, asserting that the industry should be ‘proud of low fatality rates’. These participants felt the public fails to comprehend that risk is a part of life and that statistical data on fatalities are often inaccessible or misinterpreted by lay audiences. What outsiders see as a problem of reality; is merely a problem of public perception for these stakeholders. This finding is observed within other industries where technical risk assessments often diverge from lay interpretations.^27^ This perception gap aligns with a broader phenomenon where risk is not solely about interpreting numerical data but is shaped by emotional and cultural factors.^28^, ^29^

Defining acceptable risk is complex, with no universal consensus. Sports risk perception studies often benchmark acceptable risk against everyday risks.^29^ When asked about comparative risks, interviewees suggested that horses are just as likely to be injured in the field as on the track, reflecting a perception that risk is an inescapable feature of equine life. Pearson et al. calculated that if risk at home were equivalent to the Grand National's fatality risk, a trainer with 40 horses would have a catastrophic injury on average every 10 min.^3^ This suggests even amongst industry insiders there may be misperceptions about relative risk.

High‐risk activities are not always deemed unacceptable, but risk perceptions are influenced by whether the risk is voluntarily accepted or imposed.^30^ People tend to accept higher voluntary risks, such as in sports, compared with imposed risks, such as those encountered in the workplace.^31^ Sensation‐seeking behaviour helps explain this distinction; the excitement and thrill associated with high‐risk sports often drive participation, despite significant risk. Risks that are imposed, such as those encountered in car accidents or the workplace, are generally seen as less acceptable and attract more scrutiny.^32^

Only one participant in the present study acknowledged the horses' lack of choice and agency in racing. In contrast, this is a substantial public concern.^3^, ^4^, ^9^ This difference may help explain the varying perceptions of acceptable risk between industry stakeholders and the wider public.

Some participants felt that although race day presents risks to horses, Thoroughbreds only spend a small fraction of their time on course. Participants suggested that public attention is misdirected towards race day, instead should be focused on the daily risks and welfare compromises horses experience at home. This concept of ‘frontstage and backstage’ has been explored in, for example, Winter and Frew.^33^

A recent International Equestrian Federation (FEI) framework explicitly recognises that the short time horses spend at competition poses heightened risk and is the largest threat to their welfare. Within this framework, non‐competition time is referred to as ‘the other 23 hours’.^34^

Participants in this study differed in some ways from the broader racing population. For example, this study cohort was gender‐balanced, whereas the racing industry is predominantly male‐dominated.^35^ This difference in gender representation could have an important influence on the power dynamics within the industry, as men and women perceive risk differently. Veterinarians and female participants perceived risks more acutely, aligning with literature that suggests women often perceive situations as more dangerous than men.^36^ Similarly, those directly involved with risks such as vets tend to estimate greater danger than those distanced.^37^ In contrast, educated white males, half of the interviewees, generally have lower risk perceptions,^27^ which may contribute to their more accepting attitudes towards racing risks.

Finucane et al. reported that individuals with a strong personal association with an activity tend to perceive it as less risky.^29^ All study participants self‐described as passionate racing enthusiasts. Eleven out of twelve participants were of rural background, an additional factor associated with a higher tolerance for risky behaviours.^38^ Affiliation bias, observed in other high‐risk sports such as motor racing, may also influence perceptions.^39^ Additionally, the ‘risk‐tolerant’ mindset observed in equestrians can be counterproductive to implementing a safety culture.^40^, ^41^

Parallels can be drawn between stakeholders' views on addressing perceived risk in the US and Australian racing industries. The Horse Racing Integrity and Safety Authority (HISA) was established in the US in response to public scrutiny and threats to SLO.^42^ HISA's regulatory measures represent a proactive approach to risk management but have faced industry backlash.^43^ Australia's focus on media engagement has improved public relations but has failed to address risk, welfare concerns, or protect the sport's SLO, leading to a ban on jumps racing in five states.^44^ These comparisons suggest that committed industry action is key for long‐term solutions.^45^

The tension between progress and industry preservation was found across stakeholder accounts. Participants were caught between a desire to improve safety and move with the times, while acknowledging the sports cultural heritage with a tendency towards inertia. The theme of ‘Balance between tradition and progress on reducing risks’ encapsulates this idea that reform is sometimes shaped and stifled by the past. One participant (V1) described racing as part of ‘Merry Old England’ reflecting this link between sport and historic imagery.

Stakeholders frequently linked progress and future developments to the past, portraying racing as slow to evolve, and at times defensive or reactive. Honesty and transparency were proposed by participants to be a tool to combat this and motion progress. One regulator described (R1) ‘nailing your evidence and stats’ and outlining the ‘reality’ as a further solution. However, other participants described deeper cultural challenges beyond communication and credibility. Several participants spoke of ‘entrenched attitudes’, and a reluctance to embrace change, a mentality steeped in generational knowledge. Indeed, Cassidy notes that history and heritage may be anchors to the past and hindrances to innovation.^46^, ^47^ An overly rooted attachment to the past may stifle for evidence‐based practice—a challenge observed in other sports.^48^

Beyond historical ties, participants reflected on what they saw as a culture of insularity within racing. V1 noted racing has had an ‘above everything mentality’ in the past. Interviewees referred to an echo chamber within the racing community, in which beliefs and attitudes are reinforced rather than challenged. This may be conducive to institutional attenuation, a phenomenon in which institutional processes diminish awareness or perceived importance of risks and regulatory needs.^10^, ^49^ Similarly, ‘groupthink’ where entrenched norms can obscure the need for improvements and remain unchallenged may explain this pattern. Cognitive dissonance may also play a role, where changes that conflict with established beliefs, further embedding existing attitudes.^38^ In the context of this study, these dynamics may shape and perpetuate perceptions held by stakeholders interviewed. In this sense, conversations around risk and progress appear to be less about mitigation measures and communication strategies but rather about the need for a cultural shift within the industry.

In engaging with risk, stakeholders often shifted responsibility across various actors within the industry with attribution to several different parties, including jockeys, trainers, owners, and horses themselves. This dispersal of blame may hinder cohesive efforts to improve safety and welfare standards. These factors identified show an engagement with risk; however, they are demonstrative of a fragmented approach, consistent with attribution theory in which the industry fails to take collective accountability and rather disseminates responsibility to other parties.^10^, ^11^ Further exploration of blame culture within the racing industry is warranted, but beyond the scope of this study.

Participants noted the inherent fragility of Thoroughbreds as a significant factor in race‐related injuries. McManus found the discursive construction of Thoroughbreds to be contextual, ranging from horses characterised as strong and resilient athletes, to breakable, injury prone animals.^5^ One participant's comment, ‘They're kind of a fundamentally fragile animal’, encapsulates this belief, perhaps speaking to an industry‐wide acceptance of the inevitability of injuries. This perspective may contribute to a fatalistic attitude which could hinder proactive risk management.

Participants opined that social media, generational differences, and an increasingly urbanised society were reasons for differing attitudes amongst the public and the racing industry. In contrast to the metropolitan public, participants frequently noted their rural backgrounds and pragmatic relationship with animals as the basis for a more accepting approach to animal life and death. Stakeholders further noted that less hands‐on experience with animals was responsible for a divergence of industry and public views.

An inherent distrust of institutions and a changing relationship with governance, fuelled by a culture of accountability, was thought to be further responsible for the industry–public chasm. Patterson and Hodge reported a similar generational attribution, finding that concerns for racehorse injuries increased amongst younger survey respondents.^9^ They noted that for the racing industry to engage younger viewers, concerns of catastrophic injuries would have to be assuaged. The same study highlighted social media as a contributory factor in changing attitudes towards racing. Stakeholders readily identified causative factors explaining the difference between industry and public perspectives towards risk. Engaging with these factors represents a step towards ‘moral reframing’, which requires engaging and recognising values of opposing viewpoints.^3^ In recognising that equine welfare is at the heart of both industry and public concerns, a common goal is established in the face of various factors that stakeholders believed separated their opinions from the public's. Integrating additional insights from the science of human behaviour change and social theory would support progress through an interdisciplinary approach and an embrace of systems thinking.^49^ ^,^ ^50^ This would further distance the industry from a didactic model based on a one‐way transfer of knowledge, an approach that may be a hindrance to further industry growth and sustainability.^3^

Twelve participants were recruited from thirteen approached, likely reflecting existing professional relationships with the researcher. While this familiarity helped build trust and supported open discussion, it may also have influenced how some responses were framed.

The interview guide covered a wide range of topics. While this allowed for a broad exploration of views, time constraints in some interviews may have limited how deeply certain issues were discussed. The flexible format, however, meant participants could focus on the areas they felt were most relevant.

Despite the higher risk associated with jumps racing, this study did not differentiate between National Hunt and Flat.^51^ Investigating the perspectives of stakeholders from other prominent racing nations could provide further context as to how social and cultural factors influence risk perceptions in racing. Future research should include other stakeholders, in particular trainers, whose role in risk management was repeatedly highlighted by interviewees.

## 5 CONCLUSIONS

This study has explored how United Kingdom and Irish racing stakeholders' risk perceive and approach race day risk management. Participants framed risk in varying ways, often centring on managing public narratives and reflecting on internal practices. Stakeholders recognised a slow pace of change and echo chamber dynamics in a fragmented approach to safety. While public opinion plays a powerful role in shaping racing's future, the responsibility and resources to action change lie with key stakeholders. To move forward, the industry must address internal divisions, identify shared goals, and engage with science to safeguard equine welfare and maintain public support.

## FUNDING INFORMATION

Not applicable.

## CONFLICT OF INTEREST STATEMENT

The authors declare no conflicts of interest.

## AUTHOR CONTRIBUTIONS

**Jessie McCarthy:** Conceptualization; methodology; software; investigation; data curation; validation; formal analysis; writing – review and editing; writing – original draft. **Heather A. Cameron‐Whytock:** Writing – review and editing; writing – original draft; formal analysis; validation. **Euan D. Bennet:** Conceptualization; methodology; supervision; validation; formal analysis; project administration; writing – review and editing; writing – original draft.

## DATA INTEGRITY STATEMENT

Jessie McCarthy had full access to all the data in the study and is responsible for data integrity and accuracy of the analysis.

## ETHICAL ANIMAL RESEARCH

Ethical approval for the study involving human participants was granted by the University of Glasgow, College of Medical, Veterinary and Life Sciences ethics board, application number 200230351.

## INFORMED CONSENT

Human participants of the study gave explicit informed consent for their participation.

## Supporting information


**Data S1.** Interview guide.

## Data Availability

The data that support the findings of this study are available upon reasonable request from the corresponding author. Open data sharing is not applicable to this article due to the qualitative nature of the data in this study.

## Endnote

^1^
Nvivo version 14, QSR International.
