# Supplementary material for: Striking a balance: Stakeholder perceptions of risk in horse racing
Source: Equine Vet J. 2025 Jul 7;58(3):814–23. doi: 10.1111/evj.14561 (PMC13041589; doi:10.1111/evj.14561)
Supplement: Supplementary file 1 — Data S1. Interview guide. [file EVJ-58-814-s001.pdf]

Data S1: Interview guide

This is the interview guide which was used for all semi-structured interviews conducted for the study reported in 'Striking a balance: stakeholder perceptions of risk in horse racing'. These questions served as a starting point for each interview, with further questions and discussions invoked based on where the initial answers to these guide questions took the conversations.

Introduction and Participant Background:

1. Can you describe your involvement in the racing industry ?
- How long have you been involved in the racing industry?
- What initially drew you to this industry?

Understanding Risk in Horse Racing:

2. How would you define "risk" within the context of horse racing?
3. Can you elaborate on the specific risks associated with horse racing as you perceive them?

Comparing Risk Perception and Public Acceptance:

4. From your perspective, how risky is jumps racing compared to other sports?

Media Influence and Perspectives:

5. How do you perceive the role of the media in shaping public perception of horse racing?
6. Do you think the media accurately represent horse racing?
   - Should the media adjust their messaging for audiences who are not regular Racegoers?
   - What strategies could the media employ to better communicate the risks and safety concerns of horse racing to the broader public?
7. How significant is it for the industry to consider viewpoints from individuals not directly involved with horses?
8. Can you provide examples of instances where opinions from people outside the industry were either valued or disregarded within the racing community?

Safety Measures and Industry Confidence:

9. How do you perceive recent efforts to enhance safety at racecourses?
10. When incidents such as horses dying during races, occur, how do you personally react, do you think this compares to casual racegoers and those outside racing?
11. Do you foresee that implementing additional safety measures might impact the enjoyment of horse racing? If so, how?

Understanding Risk Perception and Safety Measures in Racing:

12. Some people say that safety in horse racing is better than ever before. What do you think about this?
- Can you tell me what changes have been made in horse racing to make it safer?
- Do you think people who watch horse racing know about what's being done to make it safer for the horses and the riders?

Addressing Public Concerns and Shared Values:

13. Can you give examples of how the horse racing industry can show that they care about the safety of everyone involved?
14. Do you think the horse racing industry does a good job of talking to people about the safety of the horses and the riders?
15. Have you seen or heard of any ways the horse racing industry talks to people about safety that you think work well?
16. What do you think the horse racing industry could do better to talk to people about safety?

Addressing Misconceptions About Risk:

17. It's often suggested that horses could sustain injuries in the field just as easily as they do on the track. How do you view this argument?

Impact of Safety Measures on Racing Identity:

18. There's a belief that prioritizing safety in jumps racing may dilute its identity as a sport characterized by toughness and perseverance. How do you perceive this viewpoint?

Miscellaneous:

19. Are there any challenges you can think of that the horse racing industry might face when they try to talk to people about safety?
